# Supplementary material for: Comprehensive Genomics Investigation of Neboviruses Reveals Distinct Codon Usage Patterns and Host Specificity
Source: Microorganisms. 2024 Mar 29;12(4):696. doi: 10.3390/microorganisms12040696 (PMC11052288; doi:10.3390/microorganisms12040696)
Supplement: Supplementary file 1 [file microorganisms-12-00696-s001.zip › microorganisms-2927793-supplementary.pdf]

Supplementary Tables

Table S1. A summary of metadata of complete genome assembly of different NeVs.

NeVs	Scaffold L50	Scaffold N50	Contig L50	Contig N50	Assembly Level
BoNeV/Nebraska/2002/USA/NC_004064	1	7453	1	7453	Complete
BoNeV/TCG-14/2003/JPN/NC_006875	1	7453	1	7453	Complete
BoNev/Newbury1/2005/UK/NC_007916	1	7454	1	7454	Complete
BoNeV/M3641/2011/HUN/JX018212	1	7453	1	7453	Complete
BoNev/Kirklareli/2012/TUR/NC_030793	1	7484	1	7484	Complete
BoNeV/Mukti/2016/IND/MN241817	1	7484	1	7484	Complete
BoNeV/LZB-1/2017/CHN/MG599036	1	7453	1	7453	Complete
YAKNeV/NRG-17/2017/CHN/MK452013	NA	NA	NA	NA	Complete
BoNeV/YLA-2/2017/CHN/MH718886	1	7460	1	7460	Complete
YAKNeV/HY1-2/2018/CHN/MK452012	NA	NA	NA	NA	Complete
BoNeV/11/2021/CHN/OP272996	NA	NA	NA	NA	Complete
BoNeV/11/2021/CHN/OP272997	NA	NA	NA	NA	Complete
BoNeV/11/2021/CHN/OP272998	NA	NA	NA	NA	Complete
BoNeV/11/2021/CHN/OP272999	NA	NA	NA	NA	Complete
BoNeV/11/2021/CHN/OP273000	NA	NA	NA	NA	Complete

Table S2. A summary of codon usage (per thousand nucleotides) for the conserved regions of VP1 and VP2 genes of different NeVs.

Codons	JX018212	KT119483	MG599036	MH718886	MK452012	MK452013	MN241817	NC_004064	NC_006875	NC_007916	NC_030793	OP272996	OP272997	OP272998	OP272999	OP273000
TTT	9.4	8.7	9.4	9.4	14.9	11.7	7.9	10.2	12.5	12.5	8.7	9.4	7.1	9.4	12.6	10.2
TTC	17.2	15.7	14.9	16.4	14.9	16.4	17.4	16.4	14.8	16.4	15.7	15.0	17.3	15.0	11.0	12.6
TTA	1.6	3.2	0.8	4.7	10.2	4.7	3.2	3.1	3.9	3.9	3.2	0.8	0.8	0.8	0.8	1.6
TTG	18.0	11.8	15.6	14.1	16.4	19.5	12.6	14.1	13.3	18.0	11.8	10.2	10.2	10.2	14.2	13.4
CTT	12.5	20.5	17.2	17.2	15.6	14.1	19.7	16.4	16.4	18.0	20.5	16.5	15.0	16.5	18.1	18.1
CTC	20.3	33.1	16.4	16.4	18.0	19.5	30.8	16.4	14.1	18.0	33.1	36.2	37.8	36.2	33.9	33.1
CTA	7.8	3.2	8.6	4.7	7.8	7.8	1.6	9.4	4.7	4.7	3.2	6.3	7.1	6.3	3.2	1.6
CTG	21.9	18.1	21.9	24.2	13.3	15.6	18.9	21.9	27.3	20.3	18.1	15.0	14.2	15.0	16.5	16.5
ATT	14.8	11.0	9.4	10.9	20.3	17.2	18.9	11.7	14.8	13.3	11.0	18.9	19.7	18.9	11.8	10.2
ATC	23.4	29.9	28.9	28.9	18.8	20.3	26.8	25.8	25.8	25.8	29.9	29.9	29.1	29.9	29.1	34.6
ATA	12.5	9.4	11.7	10.2	8.6	9.4	10.3	10.9	10.2	10.2	9.4	9.4	10.2	9.4	10.2	7.9
GTT	16.4	22.8	17.2	18.8	29.7	31.3	19.7	17.2	19.5	21.1	22.8	17.3	15.0	17.3	22.0	15.7
GTC	29.7	31.5	31.3	28.1	26.6	28.9	33.1	31.3	28.1	23.4	31.5	31.5	32.3	31.5	31.5	33.1
GTA	0.8	2.4	4.7	6.3	3.9	4.7	1.6	2.3	4.7	4.7	2.4	0.8	0.8	0.8	2.4	5.5
GTG	36.7	26.8	35.2	31.3	28.1	25.8	24.4	35.2	31.3	35.9	26.8	30.7	31.5	30.7	29.9	26.8
TCT	14.8	11.8	8.6	10.9	12.5	18.0	11.0	10.2	12.5	14.8	11.8	12.6	12.6	12.6	12.6	11.0
TCC	21.9	18.1	26.6	24.2	19.5	14.9	18.9	25.0	24.2	27.3	18.1	20.5	20.5	20.5	16.5	20.5
TCA	18.8	7.9	12.5	13.3	20.3	23.5	4.7	17.2	12.5	11.7	7.9	10.2	9.4	10.2	10.2	10.2
TCG	9.4	14.2	12.5	12.5	9.4	7.8	17.4	10.2	14.1	8.6	14.2	10.2	11.0	10.2	12.6	14.2
AGT	6.3	4.7	7.0	7.0	8.6	8.6	4.7	7.8	9.4	10.2	4.7	1.6	3.2	1.6	5.5	3.2
AGC	13.3	9.4	11.7	12.5	7.8	8.6	8.7	10.2	10.9	7.0	9.4	7.9	7.1	7.9	8.7	7.9

CCT	11.7	9.4	16.4	15.6	19.5	15.6	13.4	9.4	10.9	14.8	9.4	11.8	12.6	11.8	15.0	13.4
CCC	25.8	24.4	25.0	22.7	22.7	26.6	22.1	29.7	31.3	28.9	24.4	25.2	24.4	25.2	23.6	23.6
CCA	21.1	12.6	23.5	19.5	17.2	16.4	14.2	23.4	18.8	17.2	12.6	12.6	13.4	12.6	9.4	13.4
CCG	9.4	12.6	7.8	12.5	7.8	7.8	10.3	7.0	8.6	8.6	12.6	12.6	11.8	12.6	11.0	8.7
ACT	18.8	15.0	15.6	10.9	15.6	14.1	17.4	14.1	9.4	16.4	15.0	13.4	12.6	13.4	15.7	13.4
ACC	24.2	33.1	25.0	29.7	26.6	25.8	29.2	28.1	28.9	33.6	33.1	29.9	31.5	29.9	27.6	34.6
ACA	24.2	11.8	21.1	21.9	21.9	25.8	18.1	23.4	20.3	17.2	11.8	17.3	18.9	17.3	19.7	14.2
ACG	11.7	18.9	16.4	14.8	9.4	7.0	14.2	13.3	17.2	10.2	18.9	16.5	15.0	16.5	18.1	18.9
GCT	21.1	25.2	20.3	23.4	25.8	26.6	22.9	21.1	22.7	22.7	25.2	19.7	16.5	19.7	25.2	12.6
GCC	41.4	39.4	39.1	40.6	38.3	35.2	47.3	42.2	40.6	34.4	39.4	38.6	40.9	38.6	31.5	44.1
GCA	21.1	23.6	15.6	19.5	28.1	28.9	22.9	19.5	20.3	25.0	23.6	24.4	25.2	24.4	22.8	26.8
GCG	14.8	21.3	25.8	19.5	12.5	13.3	16.6	18.0	18.8	18.0	21.3	23.6	22.8	23.6	27.6	25.2
TAT	9.4	11.0	12.5	11.7	15.6	14.1	6.3	10.2	10.9	8.6	11.0	9.4	10.2	9.4	8.7	7.1
TAC	16.4	16.5	15.6	14.8	13.3	17.2	20.5	15.6	14.8	16.4	16.5	17.3	16.5	17.3	19.7	22.8
CAT	7.8	5.5	3.1	6.3	10.9	3.9	6.3	8.6	4.7	7.8	5.5	3.9	5.5	3.9	7.1	4.7
CAC	16.4	16.5	22.7	18.0	18.0	20.3	16.6	15.6	19.5	19.5	16.5	19.7	18.1	19.7	15.0	19.7
CAA	7.0	7.9	10.9	10.2	16.4	16.4	8.7	10.9	9.4	10.2	7.9	7.1	7.1	7.1	10.2	7.1
CAG	17.2	20.5	13.3	14.1	11.7	13.3	20.5	12.5	14.1	14.1	20.5	21.3	21.3	21.3	16.5	22.0
AAT	11.7	11.8	16.4	17.2	14.9	18.8	12.6	23.4	18.0	18.8	11.8	15.7	16.5	15.7	9.4	9.4
AAC	31.3	17.3	29.7	26.6	24.2	21.9	17.4	20.3	25.0	27.3	17.3	16.5	15.7	16.5	21.3	22.0
AAA	8.6	7.1	9.4	10.2	16.4	16.4	7.9	11.7	12.5	14.8	7.1	7.1	7.1	7.1	4.7	7.9
AAG	26.6	22.8	25.8	25.8	21.1	20.3	23.7	23.4	23.4	18.8	22.8	27.6	26.8	27.6	28.3	22.0
GAT	23.4	18.9	14.9	17.2	26.6	27.4	26.0	18.8	19.5	22.7	18.9	20.5	20.5	20.5	22.0	21.3
GAC	25.0	40.2	33.6	32.0	27.4	25.0	31.5	30.5	30.5	27.3	40.2	37.8	37.8	37.8	33.9	32.3
GAA	12.5	13.4	14.9	14.8	18.0	18.8	10.3	13.3	10.2	7.8	13.4	7.1	7.1	7.1	14.2	11.8
GAG	25.0	26.8	21.9	23.4	18.0	18.8	30.0	23.4	26.6	28.1	26.8	30.7	30.7	30.7	26.0	29.9
TGT	5.5	3.2	3.1	7.0	3.1	3.1	4.7	6.3	9.4	5.5	3.2	7.1	6.3	7.1	7.1	3.2
TGC	9.4	8.7	13.3	7.8	5.5	5.5	7.1	8.6	5.5	8.6	8.7	3.9	4.7	3.9	4.7	7.9
CGT	4.7	9.4	5.5	7.0	5.5	4.7	8.7	7.0	5.5	6.3	9.4	7.1	7.1	7.1	8.7	9.4
CGC	19.5	15.0	16.4	15.6	12.5	12.5	15.0	16.4	18.0	14.8	15.0	16.5	16.5	16.5	18.9	13.4
CGA	3.1	2.4	1.6	3.1	4.7	4.7	5.5	3.1	3.1	1.6	2.4	6.3	6.3	6.3	3.2	5.5
CGG	9.4	14.2	7.8	9.4	9.4	9.4	12.6	10.9	10.2	12.5	14.2	11.0	11.0	11.0	13.4	14.2
AGA	3.1	3.2	4.7	3.1	8.6	9.4	3.2	4.7	5.5	4.7	3.2	1.6	1.6	1.6	2.4	3.2
AGG	8.6	11.0	9.4	7.8	7.0	7.8	8.7	7.8	7.0	8.6	11.0	9.4	10.2	9.4	9.4	9.4
GGT	15.6	29.1	14.9	13.3	22.7	21.1	30.8	10.2	9.4	14.8	29.1	28.3	27.6	28.3	24.4	26.0
GGC	36.7	27.6	33.6	38.3	30.5	30.5	21.3	43.8	43.0	36.7	27.6	28.3	28.3	28.3	28.3	29.1
GGA	8.6	8.7	5.5	7.0	12.5	13.3	10.3	4.7	4.7	6.3	8.7	8.7	5.5	8.7	10.2	5.5
GGG	24.2	26.0	27.4	25.8	16.4	16.4	29.2	25.8	26.6	25.8	26.0	26.8	29.9	26.8	26.8	29.9
ATG	25.0	22.0	22.7	24.2	22.7	21.9	22.1	25.0	25.0	22.7	22.0	24.4	24.4	24.4	22.8	24.4
TGG	15.6	22.0	15.6	15.6	15.6	15.6	22.1	15.6	15.6	16.4	22.0	22.0	22.0	22.0	22.0	22.0

Table S3. A summary of codon usage per thousand nucleotides for the non-conserved regions of VP1 and VP2 genes of NeVs.

Codons	JX018212	KT119483	MG599036	MH718886	MK452012	MK452013	MN241817	NC_004064	NC_006875	NC_007916	NC_030793	OP272996	OP272997	OP272998	OP272999	OP273000
TTT	13.0	9.4	15.6	12.1	17.3	21.6	12.0	13.0	15.6	15.6	9.4	12.8	15.3	12.8	9.4	9.4
TTC	19.0	17.9	15.6	19.0	16.4	14.7	14.6	19.0	19.0	15.6	17.9	11.9	9.4	11.9	17.0	17.0
TTA	6.1	0.0	5.2	3.5	5.2	8.6	0.9	0.9	2.6	5.2	0.0	0.9	2.6	0.9	0.0	2.6
TTG	19.0	12.8	21.6	20.8	19.0	17.3	10.3	18.2	21.6	22.5	12.8	7.7	7.7	7.7	8.5	10.2
CTT	14.7	14.5	13.8	18.2	13.0	17.3	18.0	20.8	15.6	16.5	14.5	21.3	21.3	22.1	17.9	14.5
CTC	26.0	31.5	18.2	19.9	19.0	14.7	24.8	20.8	26.0	23.4	31.5	22.1	23.0	21.3	25.6	26.4
CTA	4.3	6.8	7.8	4.3	7.8	7.8	4.3	7.8	8.7	6.9	6.8	6.0	3.4	6.0	7.7	7.7

CTG	22.5	15.3	26.0	25.1	19.9	19.0	18.0	23.4	18.2	19.9	15.3	22.1	22.1	22.1	19.6	17.9
ATT	21.6	18.7	24.2	20.8	19.0	19.0	17.1	21.6	26.8	16.5	18.7	18.7	15.3	18.7	17.0	17.0
ATC	21.6	25.6	19.0	23.4	20.7	19.0	26.5	22.5	18.2	23.4	25.6	23.0	26.4	23.0	25.6	29.8
ATA	6.1	10.2	5.2	2.6	13.8	13.8	11.1	4.3	3.5	7.8	10.2	11.1	11.1	11.1	11.9	11.9
GTT	16.5	19.6	16.4	16.5	25.1	23.3	15.4	17.3	21.6	22.5	19.6	21.3	21.3	21.3	12.8	14.5
GTC	17.3	25.6	19.9	17.3	11.2	13.8	30.8	17.3	19.0	13.9	25.6	29.8	29.8	29.8	33.2	32.4
GTA	6.9	2.6	6.1	2.6	3.5	5.2	4.3	6.1	10.4	4.3	2.6	2.6	3.4	2.6	2.6	2.6
GTG	33.8	19.6	32.0	39.8	25.9	25.9	19.7	35.5	28.6	31.2	19.6	21.3	20.4	21.3	22.1	18.7
TCT	7.8	7.7	6.9	8.7	14.7	11.2	10.3	7.8	11.3	6.9	7.7	7.7	8.5	7.7	8.5	7.7
TCC	21.6	14.5	27.7	22.5	19.0	19.0	15.4	24.2	24.2	22.5	14.5	17.0	17.0	17.0	15.3	16.2
TCA	14.7	11.9	14.7	15.6	19.9	21.6	10.3	17.3	16.5	15.6	11.9	13.6	13.6	13.6	10.2	12.8
TCG	13.9	12.8	13.0	10.4	10.4	11.2	12.0	7.8	7.8	11.3	12.8	12.8	12.8	12.8	11.9	9.4
AGT	9.5	8.5	6.1	5.2	10.4	10.4	9.4	10.4	8.7	7.8	8.5	11.9	9.4	11.9	8.5	9.4
AGC	10.4	15.3	13.8	13.0	10.4	13.8	11.1	10.4	13.0	13.0	15.3	7.7	9.4	7.7	12.8	15.3
CCT	20.8	8.5	13.8	14.7	16.4	20.7	15.4	20.8	19.9	16.5	8.5	15.3	12.8	16.2	14.5	17.0
CCC	22.5	30.7	26.0	25.1	28.5	22.5	27.4	18.2	17.3	24.2	30.7	28.1	30.7	27.3	31.5	26.4
CCA	20.8	23.0	15.6	14.7	25.9	25.9	20.5	19.9	21.6	18.2	23.0	16.2	17.0	16.2	21.3	17.0
CCG	13.0	14.5	19.0	20.8	9.5	11.2	13.7	16.5	14.7	15.6	14.5	16.2	15.3	16.2	13.6	16.2
ACT	13.9	18.7	11.2	9.5	13.8	12.1	12.8	11.3	9.5	7.8	18.7	17.9	17.0	17.9	20.4	21.3
ACC	19.0	34.9	27.7	29.4	21.6	22.5	38.5	25.1	26.0	25.1	34.9	31.5	33.2	31.5	31.5	34.1
ACA	7.8	17.0	7.8	10.4	16.4	14.7	18.0	7.8	11.3	8.7	17.0	18.7	16.2	18.7	11.1	8.5
ACG	16.5	17.0	13.8	14.7	11.2	8.6	15.4	16.5	15.6	18.2	17.0	10.2	11.9	10.2	17.9	16.2
GCT	19.0	24.7	22.5	20.8	23.3	25.1	18.0	20.8	13.9	22.5	24.7	25.6	25.6	25.6	19.6	25.6
GCC	48.5	47.7	36.3	43.3	29.4	27.7	53.1	43.3	47.6	45.9	47.7	48.6	48.6	48.6	51.1	46.0
GCA	13.0	20.4	14.7	16.5	15.6	17.3	25.7	10.4	10.4	13.0	20.4	22.1	22.1	22.1	21.3	18.7
GCG	9.5	21.3	11.2	8.7	5.2	4.3	20.5	13.0	13.0	11.3	21.3	22.1	21.3	22.1	20.4	24.7
TAT	4.3	10.2	8.7	10.4	13.8	12.1	10.3	6.1	8.7	6.1	10.2	6.8	6.8	6.8	8.5	7.7
TAC	18.2	12.8	13.0	11.3	9.5	13.0	12.8	15.6	13.0	15.6	12.8	17.0	17.9	17.0	14.5	15.3
CAT	6.9	7.7	11.2	7.8	7.8	6.9	6.0	8.7	10.4	10.4	7.7	3.4	3.4	3.4	8.5	6.8
CAC	14.7	10.2	8.7	12.1	12.1	12.1	10.3	11.3	8.7	11.3	10.2	14.5	13.6	14.5	11.1	10.2
CAA	18.2	11.1	14.7	15.6	23.3	23.3	11.1	19.9	14.7	21.6	11.1	12.8	12.8	12.8	14.5	13.6
CAG	23.4	21.3	25.1	26.0	17.3	18.2	23.1	21.6	26.8	20.8	21.3	19.6	19.6	19.6	19.6	19.6
AAT	18.2	13.6	18.2	15.6	15.6	20.7	13.7	15.6	19.9	15.6	13.6	16.2	15.3	16.2	25.6	13.6
AAC	19.0	29.0	20.8	22.5	22.5	19.0	28.3	19.0	18.2	19.0	29.0	28.1	29.8	28.1	20.4	28.1
AAA	10.4	7.7	10.4	12.1	15.6	17.3	8.6	9.5	13.9	9.5	7.7	11.1	11.9	11.1	7.7	4.3
AAG	30.3	23.0	29.4	28.6	26.8	25.1	21.4	33.8	26.8	31.2	23.0	18.7	17.9	18.7	23.9	23.0
GAT	18.2	20.4	14.7	13.9	18.2	22.5	19.7	18.2	18.2	20.8	20.4	12.8	14.5	12.8	19.6	12.8
GAC	30.3	19.6	38.1	35.5	28.5	21.6	23.1	32.9	33.8	31.2	19.6	26.4	24.7	26.4	23.9	28.1
GAA	15.6	13.6	15.6	13.9	19.0	19.0	13.7	16.5	10.4	13.9	13.6	12.8	11.9	12.8	7.7	11.9
GAG	31.2	29.0	25.1	29.4	29.4	29.4	26.5	29.4	32.0	30.3	29.0	30.7	31.5	30.7	34.1	32.4
TGT	4.3	6.0	2.6	3.5	5.2	3.5	6.0	3.5	1.7	0.9	6.0	6.8	6.8	6.8	6.0	5.1
TGC	6.1	11.1	9.5	8.7	9.5	6.9	12.0	7.8	7.8	8.7	11.1	9.4	9.4	9.4	11.1	11.9
CGT	6.9	10.2	10.4	10.4	4.3	6.1	8.6	6.1	7.8	9.5	10.2	11.1	11.9	11.1	6.8	10.2
CGC	22.5	30.7	21.6	19.9	19.0	16.4	31.7	23.4	22.5	15.6	30.7	27.3	26.4	27.3	29.0	29.8
CGA	3.5	3.4	3.5	3.5	3.5	4.3	2.6	5.2	5.2	6.1	3.4	4.3	3.4	4.3	3.4	3.4
CGG	8.7	6.0	6.9	6.9	12.1	12.1	8.6	6.9	3.5	9.5	6.0	9.4	10.2	9.4	9.4	8.5
AGA	6.1	0.9	4.3	5.2	9.5	10.4	0.9	4.3	6.9	5.2	0.9	1.7	1.7	1.7	4.3	2.6
AGG	11.3	8.5	12.1	13.0	9.5	7.8	10.3	10.4	10.4	14.7	8.5	8.5	8.5	8.5	5.1	11.1
GGT	17.3	20.4	21.6	12.1	25.1	24.2	22.3	14.7	19.0	15.6	20.4	15.3	18.7	15.3	14.5	20.4
GGC	34.6	32.4	32.0	43.3	29.4	32.0	30.0	39.0	34.6	39.0	32.4	38.3	33.2	38.3	34.9	29.0
GGA	5.2	6.0	6.1	5.2	14.7	12.1	9.4	6.9	11.3	9.5	6.0	9.4	10.2	9.4	6.8	9.4
GGG	26.0	22.1	26.0	26.0	17.3	18.2	19.7	24.2	19.9	19.0	22.1	17.9	18.7	17.9	22.1	18.7
ATG	17.3	23.0	20.8	17.3	23.3	24.2	23.1	19.0	15.6	19.0	23.0	23.0	23.0	23.0	23.9	26.4

TGG	20.8	11.1	20.8	20.8	19.9	20.7	11.1	20.8	20.8	21.6	11.1	11.1	11.1	11.1	11.1	11.1

Table S4. A summary of Effective Number of Codons (ENC) in VP1 and VP2 gene of different NeVs. The GC composition at 3rd synonymous codon position (GC3s), observed ENC (oENC), expected ENC (eENC) and deviation in oENC and eENC (ΔENC) are summarized. The corresponding values for VP2 are provided in parenthesis.

NeVs	GC3s	oENC	eENC	ΔENC
BoNeV/Mukti/2016/IND/MN241817	0.63 (0.61)	52.1 (53.2)	56.6 (57.9)	4.5 (4.7)
BoNev/Kirklareli/2012/TUR/NC_030793	0.64 (0.63)	52.0 (52.7)	56.2 (57.1)	4.2 (4.4)
BoNeV/TCG-14/2003/JPN/NC_006875	0.63 (0.55)	53.2 (56.2)	56.8 (60.3)	3.6 (4.1)
BoNev/Newbury1/2005/UK/NC_007916	0.63 (0.57)	52.5 (53.1)	56.7 (59.5)	4.2 (6.4)
BoNeV/LZB-1/2017/CHN/MG599036	0.65 (0.60)	52.7 (50.3)	55.9 (58.3)	3.2 (8.0)
BoNeV/M3641/2011/HUN/JX018212	0.64 (0.56)	52.2 (50.8)	56.4 (59.9)	4.2 (9.1)
BoNeV/YLA-2/2017/CHN/MH718886	0.65 (0.59)	52.7 (47.5)	55.9 (58.7)	3.2 (11.2)
YAKNeV/HY1-2/2018/CHN/MK452012	0.54 (0.47)	56.2 (57.5)	60.2 (60.2)	4.0 (2.7)
YAKNeV/NRG-17/2017/CHN/MK452013	0.54 (0.43)	56.1 (56.5)	60.3 (59.3)	4.2 (2.8)
BoNev/Newbury1/2005/UK/NC_007916	0.63 (0.55)	53.6 (52.5)	57.0 (59.9)	3.4 (7.4)
BoNeV/11/2021/CHN/OP272996	0.64 (0.56)	51.6 (55.7)	56.4 (59.7)	4.8 (4.0)
BoNeV/11/2021/CHN/OP272997	0.65 (0.56)	51.5 (55.8)	55.9 (59.7)	4.4 (4.1)
BoNeV/11/2021/CHN/OP272998	0.64 (0.56)	51.6 (55.7)	56.4 (59.7)	4.8 (4.0)
BoNeV/11/2021/CHN/OP272999	0.64 (0.59)	52.3 (53.9)	56.4 (58.7)	4.1 (4.8)
BoNeV/11/2021/CHN/OP273000	0.67 (0.61)	51.4 (53.6)	54.7 (57.9)	3.3 (4.3)

Table S5. A summary of correlation analysis (R^2) of RSCU between different NeVs and potential hosts.

GenBank Id	*Bos taurus*	*Bubalus bubalis*	*Capra hircus*	*Ovis aries*	*Equus caballus*	*Equus asinus*	*Camelus dromedarius*
MN241817	0.34	0.28	0.27	0.31	0.33	0.31	0.32
NC_030793	0.31	0.26	0.25	0.29	0.30	0.31	0.30
NC_006875	0.48	0.43	0.43	0.34	0.49	0.38	0.47
NC_004064	0.47	0.41	0.41	0.34	0.47	0.39	0.47
MG599036	0.44	0.39	0.38	0.33	0.44	0.40	0.46
JX018212	0.50	0.43	0.43	0.36	0.50	0.40	0.47
MH718886	0.53	0.46	0.46	0.39	0.53	0.44	0.53
MK452012	0.32	0.31	0.31	0.23	0.32	0.31	0.31
MK452013	0.32	0.32	0.31	0.20	0.33	0.31	0.31
NC_007916	0.48	0.44	0.43	0.39	0.48	0.47	0.49
OP272996	0.34	0.27	0.27	0.28	0.34	0.29	0.34
OP272997	0.34	0.28	0.27	0.30	0.34	0.31	0.34
OP272998	0.34	0.27	0.27	0.28	0.34	0.28	0.34
OP272999	0.33	0.26	0.26	0.29	0.33	0.28	0.30

OP273000	0.35	0.31	0.29	0.34	0.36	0.37	0.37

Table S6. A summary of common preferred codons (out of 59 codons) in different strains of NeVs and different hosts. In the parenthesis the total number preferred codons in NeVs and hosts are provided.

GenBank Id	*B. Taurus* (28)	*B. bubalis* (26)	*C. hircus* (26)	*O. aries* (27)	*E. caballus* (27)	*E. asinus* (26)	*C. dromedarius* (26)
MN241817 (26)	21	20	20	19	21	20	20
NC_030793 (30)	23	22	22	21	23	22	22
NC_006875 (27)	20	20	20	19	20	20	19
NC_004064 (27)	21	20	20	19	21	20	20
MG599036 (26)	21	21	21	20	21	21	20
JX018212 (27)	22	21	21	20	22	21	21
MH718886 (25)	21	20	20	19	21	20	20
MK452012 (30)	20	18	19	18	19	18	18
MK452013 (33)	22	20	20	18	21	20	20
NC_007916 (26)	21	19	20	19	21	19	20
OP272996 (27)	20	18	18	17	20	18	19
OP272997 (26)	21	19	19	18	21	19	20
OP272998 (27)	20	18	18	17	20	18	19
OP272999 (24)	21	19	19	18	21	19	20
OP273000 (26)	22	20	20	19	22	20	21

Table S7. A summary of codon adaptation indices of VP1 gene of NeVs corresponding to different host.

NeVs	*B. taurus*	*B. bubalis*	*C. hircus*	*O. aries*	*E. caballus*	*E. asinus*	*C. dromedarius*
BoNeV/Nebraska/2002/USA/NC_004064	0.712	0.719	0.700	0.676	0.711	0.703	0.667
BoNeV/TCG-14/2003/JPN/NC_006875	0.711	0.716	0.697	0.673	0.708	0.699	0.662
BoNev/Newbury1/2005/UK/NC_007916	0.711	0.718	0.700	0.683	0.710	0.707	0.665
BoNeV/M3641/2011/HUN/JX018212	0.720	0.725	0.707	0.687	0.719	0.710	0.672
BoNev/Kirklareli/2012/TUR/NC_030793	0.694	0.698	0.680	0.669	0.693	0.695	0.646
BoNeV/Mukti/2016/IND/MN241817	0.700	0.703	0.685	0.674	0.698	0.696	0.650
BoNeV/LZB-1/2017/CHN/MG599036	0.707	0.711	0.692	0.674	0.705	0.697	0.661

YAKNeV/NRG-17/2017/CHN/MK452013	0.691	0.702	0.683	0.658	0.688	0.684	0.642
BoNeV/YLA-2/2017/CHN/MH718886	0.718	0.722	0.704	0.686	0.716	0.710	0.673
YAKNeV/HY1-2/2018/CHN/MK452012	0.693	0.702	0.686	0.663	0.691	0.685	0.644
BoNeV/11/2021/CHN/OP272996	0.698	0.702	0.683	0.668	0.697	0.691	0.651
BoNeV/11/2021/CHN/OP272997	0.699	0.702	0.683	0.671	0.697	0.693	0.651
BoNeV/11/2021/CHN/OP272998	0.698	0.701	0.683	0.668	0.697	0.691	0.65
BoNeV/11/2021/CHN/OP272999	0.692	0.696	0.677	0.663	0.692	0.689	0.642
BoNeV/11/2021/CHN/OP273000	0.702	0.705	0.686	0.678	0.701	0.7	0.655

Table S8. A summary of codon adaptation indices of VP2 gene of NeVs corresponding to different host.

NeVs	*B. taurus*	*B. bubalis*	*C. hircus*	*O. aries*	*E. caballus*	*E. asinus*	*C. dromedarius*
BoNeV/Nebraska/2002/USA/NC_004064	0.707	0.723	0.703	0.683	0.710	0.724	0.649
BoNeV/TCG-14/2003/JPN/NC_006875	0.709	0.717	0.700	0.679	0.707	0.694	0.646
BoNev/Newbury1/2005/UK/NC_007916	0.716	0.728	0.711	0.689	0.717	0.711	0.652
BoNeV/M3641/2011/HUN/JX018212	0.706	0.719	0.698	0.675	0.706	0.711	0.643
BoNev/Kirklareli/2012/TUR/NC_030793	0.670	0.671	0.655	0.646	0.666	0.658	0.613
BoNeV/Mukti/2016/IND/MN241817	0.673	0.675	0.659	0.653	0.671	0.668	0.615
BoNeV/LZB-1/2017/CHN/MG599036	0.720	0.728	0.709	0.685	0.719	0.707	0.660
YAKNeV/NRG-17/2017/CHN/MK452013	0.657	0.678	0.650	0.625	0.656	0.657	0.594
BoNeV/YLA-2/2017/CHN/MH718886	0.724	0.736	0.714	0.681	0.724	0.712	0.661
YAKNeV/HY1-2/2018/CHN/MK452012	0.661	0.678	0.651	0.638	0.659	0.661	0.599
BoNeV/11/2021/CHN/OP272996	0.655	0.66	0.644	0.634	0.655	0.655	0.6
BoNeV/11/2021/CHN/OP272997	0.67	0.677	0.66	0.647	0.671	0.672	0.613
BoNeV/11/2021/CHN/OP272998	0.655	0.66	0.644	0.634	0.655	0.655	0.6
BoNeV/11/2021/CHN/OP272999	0.686	0.69	0.674	0.658	0.684	0.674	0.628
BoNeV/11/2021/CHN/OP273000	0.671	0.675	0.659	0.66	0.67	0.675	0.618

Table S9. A summary of observed RCDI (oRCDI) values and expected RCDI (eRCDI) values for VP1 genes of different NeVs corresponding to different hosts. The expected RCDI (eRCDI) values for all the hosts are provided in parenthesis after their names.

	B. Taurus (1.43)	*B. bubalis* (1.54)	*C. hircus* (1.57)	*O. aries* (1.65)	*E. caballus* (1.41)	*E. asinus* (1.61)	*C. dromedarius* (1.59)
MN241817	1.13	1.17	1.18	1.19	1.14	1.17	1.17
NC_030793	1.14	1.18	1.19	1.20	1.15	1.18	1.19
NC_006875	1.09	1.11	1.12	1.18	1.09	1.15	1.13
NC_004064	1.09	1.11	1.12	1.18	1.10	1.15	1.12
MG599036	1.10	1.13	1.14	1.18	1.11	1.16	1.13
JX018212	1.08	1.11	1.11	1.16	1.09	1.13	1.11
MH718886	1.09	1.12	1.12	1.16	1.09	1.13	1.11
MK452012	1.11	1.11	1.12	1.20	1.11	1.14	1.15
MK452013	1.11	1.11	1.12	1.21	1.11	1.14	1.15
NC_007916	1.08	1.10	1.11	1.15	1.09	1.11	1.11
OP272996	1.131	1.166	1.369	1.197	1.137	1.186	1.165
OP272997	1.134	1.169	1.323	1.192	1.139	1.182	1.168
OP272998	1.131	1.166	1.369	1.197	1.136	1.186	1.165
OP272999	1.141	1.179	1.249	1.21	1.145	1.193	1.19
OP273000	1.133	1.168	1.374	1.183	1.136	1.167	1.168

Table S10. A summary of observed RCDI (oRCDI) values and expected RCDI (eRCDI) values for VP2 genes of different NeVs corresponding to different hosts. The expected RCDI (eRCDI) values for all the hosts are provided in parenthesis after their names.

	B. Taurus (1.47)	*B. bubalis* (1.57)	*C. hircus* (1.61)	*O. aries* (1.70)	*E. caballus* (1.52)	*E. asinus* (1.69)	*C. dromedarius* (1.60)
MN241817	1.34	1.39	1.39	1.42	1.35	1.40	1.42
NC_030793	1.32	1.38	1.37	1.39	1.34	1.42	1.39
NC_006875	1.29	1.30	1.31	1.47	1.29	1.33	1.37
NC_004064	1.42	1.40	1.41	1.59	1.40	1.36	1.51
MG599036	1.42	1.43	1.44	1.63	1.41	1.45	1.51
JX018212	1.44	1.45	1.47	1.67	1.44	1.46	1.56
MH718886	1.40	1.40	1.42	1.62	1.40	1.45	1.48
MK452012	1.41	1.37	1.43	1.56	1.41	1.41	1.49
MK452013	1.46	1.42	1.49	1.67	1.46	1.48	1.56
NC_007916	1.39	1.39	1.40	1.59	1.38	1.41	1.51
OP272996	1.335	1.376	1.179	1.412	1.34	1.391	1.399

OP272997	1.303	1.33	1.175	1.381	1.306	1.343	1.367
OP272998	1.335	1.376	1.192	1.412	1.34	1.391	1.399
OP272999	1.218	1.257	1.179	1.299	1.229	1.311	1.284
OP273000	1.328	1.377	1.393	1.336	1.373	1.402	1.273

Table S11. A summary of frequencies of tRNA genes in different hosts (*Bos taurus*, *Equus caballus*, and *Ovis aries*) for most preferentially used codons in NeVs.

	ALA	ARG	ASN	ASP	CYS	GLN	GLU	GLY	HIS	ILE	LEU	LYS	PHE	PRO	SER	THR	TYR	VAL
NC_030793	GCC	CGC	AAC	GAC	TGC	CAG	GAG	GGC	CAC	ATC	CTC	AAG	TTC	CCC	TCC	ACC	TAC	GTC
NC_006875	GCC	CGC	AAC	GAC	TGC	CAG	GAG	GGC	CAC	ATC	CTG	AAG	TTC	CCC	TCC	ACC	TAC	GTG
NC_004064	GCC	CGC	AAC	GAC	TGC	CAG	GAG	GGC	CAC	ATC	CTG	AAG	TTC	CCC	TCC	ACC	TAC	GTG
MG599036	GCC	CGC	AAC	GAC	TGC	CAG	GAG	GGC	CAC	ATC	CTG	AAG	TTC	CCC	TCC	ACC	TAC	GTG
JX018212	GCC	CGC	AAC	GAC	TGC	CAG	GAG	GGC	CAC	ATC	CTC	AAG	TTC	CCC	TCC	ACC	TAC	GTG
MH718886	GCC	CGC	AAC	GAC	TGC	CAG	GAG	GGC	CAC	ATC	CTG	AAG	TTC	CCC	TCC	ACC	TAC	GTG
MK452012	GCC	CGC	AAC	GAC	TGC	CAA	GAG	GGC	CAC	ATC	CTC	AAG	TTT	CCC	TCA	ACC	TAT	GTT
MK452013	GCC	CGC	AAC	GAT	TGC	CAA	GAG	GGC	CAC	ATC	TTA	AAG	TTT	CCC	TCA	ACC	TAC	GTT
NC_007916	GCC	CGC	AAC	GAC	TGC	CAG	GAG	GGC	CAC	ATC	CTC	AAG	TTC	CCC	TCC	ACC	TAC	GTG
KT119483	GCC	CGC	AAC	GAC	TGC	CAG	GAG	GGC	CAC	ATC	CTC	AAG	TTC	CCC	TCC	ACC	TAC	GTC
MN241817	GCC	CGC	AAC	GAC	TGC	CAG	GAG	GGT	CAC	ATC	CTC	AAG	TTC	CCC	TCC	ACC	TAC	GTC
OP272996	GCC	CGC	AAC	GAC	TGT	CAG	GAG	GGC	CAC	ATC	CTC	AAG	TTC	CCC	TCC	ACC	TAC	GTC
OP272997	GCC	CGC	AAC	GAC	TGC	CAG	GAG	GGC	CAC	ATC	CTC	AAG	TTC	CCC	TCC	ACC	TAC	GTC
OP272998	GCC	CGC	AAC	GAC	TGT	CAG	GAG	GGC	CAC	ATC	CTC	AAG	TTC	CCC	TCC	ACC	TAC	GTC
OP272999	GCC	CGC	AAC	GAC	TGC	CAG	GAG	GGC	CAC	ATC	CTC	AAG	TTC	CCC	TCC	ACC	TAC	GTC
OP273000	GCC	CGC	AAC	GAC	TGC	CAG	GAG	GGC	CAC	ATC	CTC	AAG	TTC	CCC	TCC	ACC	TAC	GTC
Bos taurus	AGC (30)<br>TGC (14)<br>CGC (8)<br>GGC (0)	TCG (10)<br>ACG (9)<br>CCT (8)<br>TCT (6)<br>CCG (4)<br>GCG (0)	GTT (27)<br>ATT (0)	GTC (23)<br>ATC (0)	GCA (27)<br>ACA (0)	CTG (20)<br>TTG (8)	TTC (31)<br>CTC (11)	GCC (16)<br>CCC (14)<br>TCC (10)<br>ACC (0)	GTG (16)<br>ATG (0)	AAT (16)<br>TAT (6)<br>GAT (0)<br>CAT (0)	AAG (9)<br>CAG (8)<br>CAA (6)<br>TAA (6)<br>TAG (4)<br>GAG (0)	TTT (31)<br>CTT (22)	GAA (23)<br>AAA (0)	AGG (13)<br>TGG (10)<br>CGG (4)<br>GGG (0)	GCT (20)<br>AGA (12)<br>CGA (5)<br>TGA (4)<br>GGA (2)	AGT (12)<br>TGT (9)<br>CGT (6)<br>GGT (0)	GTA (17)<br>ATA (0)	CAC (17)<br>AAC (15)<br>TAC (10)<br>GAC (0)
Total	52	37	27	23	27	28	42	40	16	22	35	53	23	27	41	27	17	42
Equus caballus	AGC (24)<br>TGC (11)	TCG (4)<br>ACG (10)	GTT (14)<br>ATT (0)	GTC (8)<br>ATC (0)	GCA (22)<br>ACA (0)	CTG (12)<br>TTG (5)	TTC (10)<br>CTC (10)	GCC (10)<br>CCC (8)	GTG (11)<br>ATG (0)	AAT (17)<br>TAT (5)	AAG (7)<br>CAG (3)	TTT (14)<br>CTT (14)	GAA (12)<br>AAA (0)	AGG (10)<br>TGG (7)	GCT (11)<br>AGA (12)	AGT (9)<br>TGT (7)	GTA (14)<br>ATA (0)	CAC (14)<br>AAC (12)

	CGC<br>(8)<br>GGC<br>(0)	CCT<br>(7)<br>TCT<br>(5)<br>CCG<br>(4)<br>GCG<br>(0)						TCC<br>(4)<br>ACC<br>(0)		GAT<br>(0)<br>CAT<br>(0)	CAA<br>(6)<br>TAA<br>(4)<br>TAG<br>(5)<br>GAG<br>(0)			CGG<br>(3)<br>GGG<br>(0)	CGA<br>(4)<br>TGA<br>(4)<br>GGA<br>(0)	CGT<br>(3)<br>GGT<br>(0)		TAC<br>(6)<br>GAC<br>(0)
Total	43	30	14	8	22	17	20	22	11	22	25	28	12	20	31	19	14	32
	ALA	ARG	ASN	ASP	CYS	GLN	GLU	GLY	HIS	ILE	LEU	LYS	PHE	PRO	SER	THR	TYR	VAL
Ovis aries	AGC<br>(29)<br>TGC<br>(16)<br>CGC<br>(8)<br>GGC<br>(0)	TCG<br>(9)<br>ACG<br>(9)<br>CCT<br>(8)<br>TCT<br>(6)<br>CCG<br>(4)<br>GCG<br>(0)	GTT<br>(20)<br>ATT<br>(0)	GTC<br>(13)<br>ATC<br>(0)	GCA<br>(28)<br>ACA<br>(0)	CTG<br>(12)<br>TTG<br>(7)	TTC<br>(29)<br>CTC<br>(13)	GCC<br>(14)<br>CCC<br>(10)<br>TCC<br>(11)<br>ACC<br>(0)	GTG<br>(12)<br>ATG<br>(0)	AAT<br>(13)<br>TAT<br>(5)<br>GAT<br>(1)<br>CAT<br>(0)	AAG<br>(10)<br>CAG<br>(5)<br>CAA<br>(6)<br>TAA<br>(6)<br>TAG<br>(4)<br>GAG<br>(0)	TTT<br>(23)<br>CTT<br>(22)	GAA<br>(24)<br>AAA<br>(0)	AGG<br>(12)<br>TGG<br>(7)<br>CGG<br>(5)<br>GGG<br>(0)	GCT<br>(14)<br>AGA<br>(15)<br>CGA<br>(4)<br>TGA<br>(4)<br>GGA<br>(0)	AGT<br>(10)<br>TGT<br>(7)<br>CGT<br>(5)<br>GGT<br>(0)	GTA<br>(19)<br>ATA<br>(0)	CAC<br>(10)<br>AAC<br>(11)<br>TAC<br>(5)<br>GAC<br>(0)
Total	53	36	20	13	28	19	42	35	12	19	31	45	24	24	37	22	19	26

Table S12. A summary of recombination events observed from multiple sequence alignment of complete genome sequences of NeVs. The recombination events that are detected by at least 2 methods, out of total seven methods used, are accounted here. The recombinants, major parents and its position on genome and the minor parents with its position on genome are provided.

Recombinant	Major Parent	Positions	Minor Parent	Positions
MG599036	JX018212	1 – 5317; 7100 – 7935	NC_007916*	5318 – 7099
NC_007916	MH718886*	1 – 5291; 7353 – 7935	NC_004064	5292 – 7352
NC_004064	NC_007916	5292 – 7915	MH718886	1 – 5291; 7916 – 7935
JX018212	MH718886	1 – 5349; 7431 – 7935	NC004064	5350 – 7430

Supplementary Figures

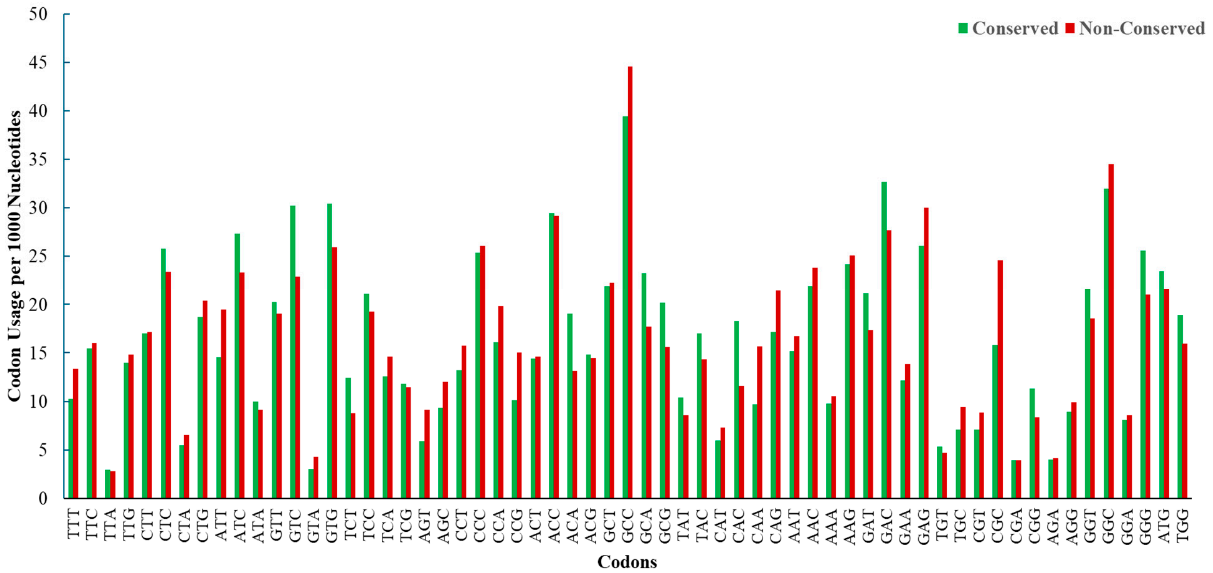

Figure S1. A depiction of an overall codon usage in conserved and non-conserved regions of VP1 and VP2 genes of NeVs.

	OP273000	NC_030793	KT119483	MN241817	OP272999	OP272996	OP272998	OP272997	MK452012	MK452013	MG599036	NC_007916	MH718886	NC_006875	NC_004064	JX018212
OP273000		88	88	89	100	90	90	91	31	29	30	31	29	29	31	31
NC_030793	92		100	89	89	89	89	89	30	30	30	30	30	29	31	30
KT119483	92	100		89	89	89	89	89	30	30	30	30	30	29	31	30
MN241817	92	96	96		89	89	89	89	30	30	30	30	30	29	31	30
OP272999	93	93	93	93		90	90	91	30	31	31	30	30	29	31	31
OP272996	93	92	92	92	93		100	99	30	29	31	31	31	30	31	31
OP272998	93	92	92	92	93	100		99	30	29	31	31	31	30	31	31
OP272997	93	92	92	92	93	100	100		30	30	31	32	31	30	31	32
MK452012	43	43	43	43	43	43	43	43		97	79	80	80	79	79	77
MK452013	43	43	43	43	43	43	43	43	96		80	80	81	80	79	78
MG599036	42	43	43	43	43	43	43	43	75	75		94	96	94	92	92
NC_007916	42	43	43	43	43	43	43	43	76	76	88		96	95	95	96
MH718886	42	43	43	43	43	43	43	43	76	76	93	92		96	93	92
NC_006875	42	43	43	42	43	43	43	43	76	76	91	93	96		92	94
NC_004064	42	43	43	43	43	43	43	43	76	76	91	94	96	97		94
JX018212	43	43	43	43	43	43	43	43	76	76	91	93	96	95	97	

Figure S2. A heatmap representation of pairwise homology of VP1 and VP2 genes of different NeVs. The lower triangle represents VP1 genes while the upper triangle represents VP2 genes.
